# Study of Hydrophilic Electrospun Nanofiber Membranes for Filtration of Micro and Nanosize Suspended Particles

**DOI:** 10.3390/membranes3040375

**Published:** 2013-11-14

**Authors:** Ramazan Asmatulu, Harish Muppalla, Zeinab Veisi, Waseem S. Khan, Abu Asaduzzaman, Nurxat Nuraje

**Affiliations:** 1 Department of Mechanical Engineering, Wichita State University, 1845 Fairmount, Wichita, KS 67260, USA; E-Mails: z.veisi@gmail.com (Z.V.); waseem_beacon@yahoo.com (W.S.K.); 2 Department of Electrical Engineering and Computer Science, Wichita State University, 1845 Fairmount, Wichita, KS 67260, USA; E-Mails: harish00309@gmail.com (H.M.); abu.asaduzzaman@wichita.edu (A.A.); 3 Department of Materials Science and Engineering, Massachusetts Institute of Technology, Cambridge, MA 02139, USA

**Keywords:** electrospun nanofiber, water filtration, hydrophilic membranes, micro and nanosize particles, filtration

## Abstract

Polymeric nanofiber membranes of polyvinyl chloride (PVC) blended with polyvinylpyrrolidone (PVP) were fabricated using an electrospinning process at different conditions and used for the filtration of three different liquid suspensions to determine the efficiency of the filter membranes. The three liquid suspensions included lake water, abrasive particles from a water jet cutter, and suspended magnetite nanoparticles. The major goal of this research work was to create highly hydrophilic nanofiber membranes and utilize them to filter the suspended liquids at an optimal level of purification (*i.e.*, drinkable level). In order to overcome the fouling/biofouling/blocking problems of the membrane, a coagulation process, which enhances the membrane’s efficiency for removing colloidal particles, was used as a pre-treatment process. Two chemical agents, Tanfloc (organic) and Alum (inorganic), were chosen for the flocculation/coagulation process. The removal efficiency of the suspended particles in the liquids was measured in terms of turbidity, pH, and total dissolved solids (TDS). It was observed that the coagulation/filtration experiments were more efficient at removing turbidity, compared to the direct filtration process performed without any coagulation and filter media.

## 1. Introduction

Polymeric nanofibers have gained much attention in the scientific community for their numerous applications in the fields of nanotechnology, biotechnology, and other related technologies [1]. Electrospinning is one of the many methods to produce various sizes and shapes of polymeric and ceramic nanofibers [2]. Due to their nanoscale diameters, the fibers exhibit a large surface area-to-volume ratio, flexibility, and fine pore texture. These properties have enhanced the importance of polymeric nanofibers in many industrial and biological applications, such as molecular filtration, tissue engineering, drug delivery, wound dressing, cosmetics, solar and fuel cells, bio and nano sensors, *etc*. [1,2,3,4]. 

Electrospinning is a relatively easy, fast, and cost-effective process of producing fibers that range from micrometers (~100 µm) to nanometers (~3 nm) [1]. Compared to other nanomanufacturing techniques, the process is very simple, whereby a drop of polymeric solution is initially held at the tip of a needle by the surface tension of the polymeric solution. When the applied voltage to the polymeric solution overcomes the surface tension, a charged jet erupts from the tip of the capillary tube, first following a linear path and then experiencing bending instability from spinning, and finally is collected on a grounded collector screen, placed at some distance from the spinneret [4,5,6,7]. 

Water treatment processes employ several types of membranes, including microfiltration (MF), ultrafiltration (UF), reverse osmosis (RO), and nanofiltration (NF) [3]. Although the RO membrane provides the highest purity of water, the NF membrane has been gaining considerable attention because of its ability to rapidly and economically remove total dissolved solids from the surface and ground water, pathogens (bacteria, virus, molds, and fungus), monovalent and multivalent anion and cations (water softening), salts, minerals, and other suspended nanoparticles [4]. The NF membrane has many other applications in various industries, such as the food and beverage, cosmetic, chemical, oil, pharmaceutical, mining, metallurgical, and textile. The pore size of the NF membrane can be as small as 1 nm in order to selectively remove larger molecules from smaller molecules and size the nanomaterials in different fractions [4,5,6,7,8]. 

A coagulant is “an agent that induces curdling or congealing”. The main purpose of using the coagulation process in wastewater treatment is to neutralize the colloids. This occurs by providing a positive charge to the negatively charged effluents [4], which results in the formation of larger particles or flocs that can be easily removed physically. In general, this is a pretreatment process prior to filtration applications, such as drinking water production and filtration of industrial wastewater. The pretreatment process enhances the efficiency of the filtration process by reducing turbidity and the color of the wastewater [9,10,11,12].

Hydrophilicity is an important issue for the wettability of membranes and development of low-pressure filters because the lower water contact angle of hydrophilic membranes will significantly reduce the capillary pressure of the filter media and increase the flow rate of the liquid and separation ability of the suspended particles. In addition, it will greatly eliminate the biofouling and clogging of the filter media during the filtration process [3,4]. The polyvinyl chloride (PVC) nanomembrane exhibits a hydrophobic nature and cannot be used easily for the filtration process. The wettability of the membrane surface depends on its microstructure and chemical nature; however, surface modification can be applied on the membrane to increase its hydrophilicity [13,14,15,16,17]. In this study, water soluble polyvinylpyrrolidone (PVP) was added into PVC at different percentages (2%, 3%, 4%, and 5%) and then electrospun to produce highly hydrophilic nanofiber membranes. The surface properties of the membranes were studied in relation to their filtration efficiency for different water suspensions.

## 2. Results and Discussion

### 2.1. Study of Hydrophilic Nanofibrous Membranes

**Figure 1:**
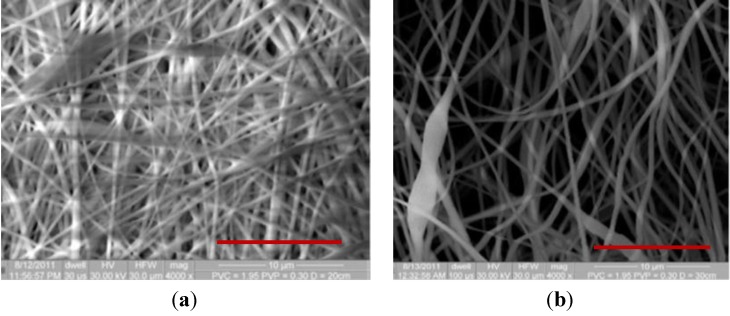
SEM images of PVC fiber with 2 wt % PVP at spinneret distance of (**a**) 20 cm and (**b**) 30 cm (scale bar is 10 µm).

**Figure 2:**
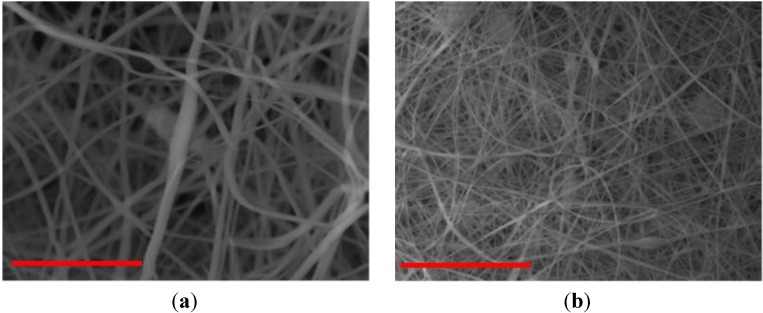
SEM images of PVC fiber with 4 wt % PVP fibers at spinneret distance of (**a**) 20 cm and (**b**) 30 cm (scale bar is 10 µm).

The nanomembranes were fabricated using the electrospinning technique. The PVC was first dissolved in the solvent DMAc by using a magnetic bar and hot plate at 65 °C for about 4 h. The concentration of PVC was kept at 15%. Electrospinning experiments were conducted at various distances (D) of 20, 25, and 30 cm, while keeping the DC voltage and pump speed constant. Fiber size and structural morphology of all membranes were analyzed using scanning electron microscopy (SEM), as shown in Figure 1. Figure 1A,B shows SEM images of electrospun fibers formed with PVC and 2% PVP at three different distances: 20, 25, and 30 cm. It can be seen in Figure 1 and Figure 2 that the increase in spinneret distance has reduced the fiber diameters. Fibers that formed at three distances are found to be at nanoscale (below 500 nm). Figure 2 shows SEM images of electrospun fibers formed with PVC and 4 wt % PVP at three different distances: 20, 25, and 30 cm. At a spinneret distance of 20 cm, the fiber diameters for 2% and 3% PVP are similar. In the case of 25 and 30 cm, the fiber diameters are reduced. A 30 cm of spinneret distance was used in the studies. From Figure 2B, it can be seen that the fibers formed are consistent throughout the membrane at nanoscale diameters of 200 to 600 nm. The fiber diameters are reduced with the increase in spinneret distance, which can be seen in Figure 2A. The diameters of electrospun fibers formed with PVC and 4 wt % and 5 wt % PVP at three different distances of 20, 25, and 30 cm are at nanoscale. Fiber diameters are very consistent throughout the membranes. 

**Table 1:** Contact angle values of polyvinyl chloride (PVC) membranes incorporated with different weight percentages of polyvinylpyrrolidone (PVP) at three spinneret distances.

PVC/PVP Fibers	Water Contact Angle (°)		
	D = 20 cm	D = 25 cm	D = 30 cm
Only PVC Fibers	136.02	135.76	135.70
Only PVP Fibers (water soluble)	0.00	0.00	0.00
PVC + 2 wt % PVP Fibers	127.14	125.26	124.23
PVC + 3 wt % PVP Fibers	118.35	116.08	105.85
PVC + 4 wt % PVP Fibers	103.03	70.95	58.51
PVC + 5 wt % PVP Fibers	21.30	20.30	16.12

In order to investigate the effect of surface hydrophilicity of the membrane on waste water filtration, polymeric membranes of PVC with varied PVP were prepared. Water filtration efficiency of the composite membrane was investigated via contact angle and turbidity methods. Table 1 and Table 2 provide the contact angle values of the electrospun membranes at three different spinneret distances. From Table 1 and Table 2, it can be seen that the water contact angle of PVC fibers without PVP was 135.70°; however, water contact angles of the PVC fibers with 2, 3, 4, and 5 wt % of PVP were 124.23°, 105.85°, 58.51°, and 16.12°, respectively. This indicates that incorporation of PVP in PVC polymer changes the surface characteristics and morphology of electrospun fibers because of the hydrophilic and soluble nature of PVP. In addition, adding PVP into PVC accelerates the formation of the Wenzel state of the fiber membranes and makes the filtration process easier [13,17,18]. Thus, permeability and filtration rates of the waste water samples were significantly high at lower water contact angles. For example, 100 mL of DI water passed through PVC and PVC + 4 wt % PVP fibers at 19 and 8 s at 25 inHg, respectively. For this reason, the electrospun fibers formed with 4 wt % PVP are considered for all filtration experiments throughout this study. Beyond 4 wt % PVP in PVC, the nanofiber membranes appear to be flimsy and fragile. Based on the following hypothesis, that the surface property changing from hydrophobic to hydrophilic is supposed to improve the filtration process (higher filtration kinetics) by making it easier and faster and preventing the blocking of the nanofiber membranes [4], the PVC nanofibers with 4 wt % PVP were evaluated in the following coagulation-filtration experiments. PVP molecules on the surface of PVC fibers may potentially leach out, so in the future studies we will investigate the potential leaching process of PVP.

**Table 2:** Water contact angle values of PVC fibers incorporated with PVP at different separation distances.

Electrospun Fibers	Water Contact Angle (°)		
	20 cm	25 cm	30 cm
PVC + 2 wt % PVP Fibers	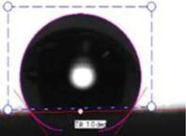	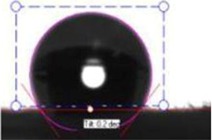	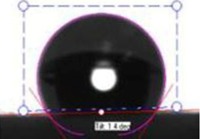
PVC + 3 wt % PVP Fibers	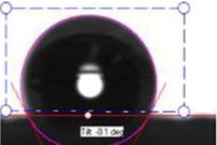	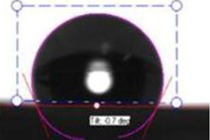	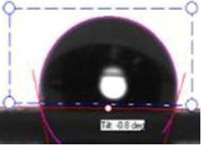
PVC + 4 wt % PVP Fibers	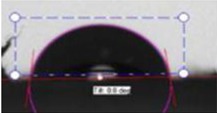	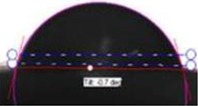	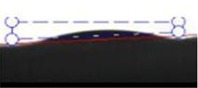
PVC + 5 wt % PVP Fibers	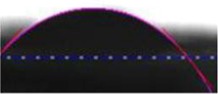	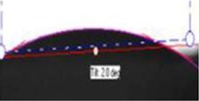	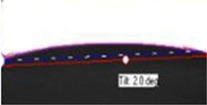

### 2.2. Coagulation-Filtration Experiments

#### 2.2.1. Lake Water

Table 3 gives the turbidity, pH, and total dissolved solids (TDS) values of the lake water as a function of Tanfloc additions after 1 h and 24 h settlements by gravity. Test results show that the turbidity was drastically reduced by increasing the Tanfloc concentrations. The initial turbidity value of the lake water was 21 nephelometric turbidity units (NTU), and one hour later, the turbidity values of the lake water were reduced to 6.4, 4.7, 3.5, 4.9, and 4.9 NTU at 5, 10, 15, 20, and 25 mg/L Tanfloc dosages, respectively. The turbidity values were further reduced to 2.6, 2.3, 1.0, 1.8, and 1.8 NTU at the same Tanfloc concentrations one day later. The residual turbidity values of the lake water were reduced about 15% after 1 h and about 25% after 24 h without any addition of the Tanfloc coagulants (only settlement due to the gravity). It was decided that the 15 mg/L of Tanfloc concentration and 1 h duration are sufficient to obtain clean water from the lake water. 

**Table 3:** Turbidity, pH, and total dissolved solids (TDS) values of lake water as function of Tanfloc additions after 1 h and 24 h settlements by gravity.

Experiments	No Coagulation			Coagulation Dosage (mg/L) (1 h)					Coagulation Dosage (mg/L) (1 day)				
	0 h	1 h	24 h	5	10	15	20	25	5	10	15	20	25
Turbidity (NTU)	21.0	17.9	15.8	6.4	4.7	3.5	4.9	4.9	2.6	2.3	1.0	1.8	1.8
pH	8.1			8.1	8.1	8.1	8.1	8.1	8.1	8.1	8.1	8.1	8.1
TDS (ppm)	480			472	480	460	464	458	472	480	460	464	458

**Figure 3:**
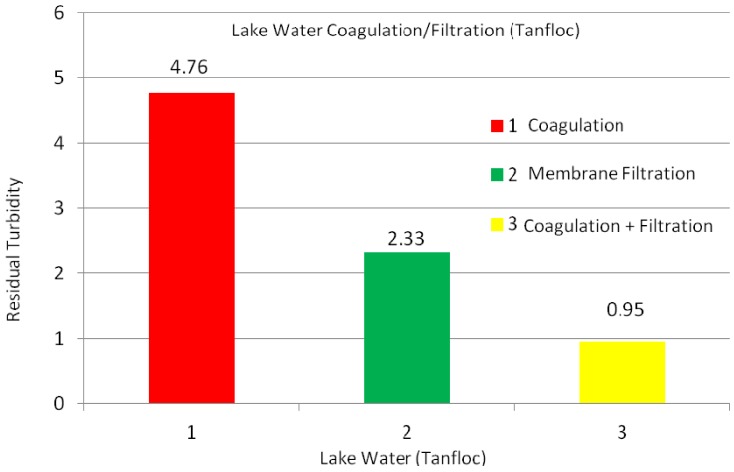
Turbidity values of lake water using Tanfloc coagulation, membrane filtration, and coagulation + filtration processes; coagulation tests conducted using 15 mg/L Tanfloc after 1 h of settlement in flask, while filtration tests performed using Buchner filter after same amount of time.

**Figure 4:**
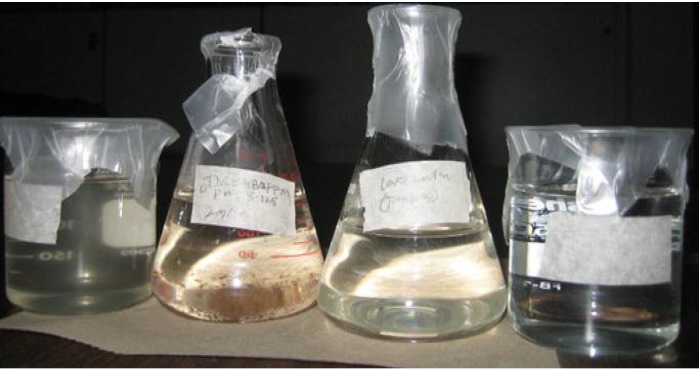
Lake water treatment processes, from left: untreated lake water, Tanfloc coagulation treatment (settlement), direct filtration, and coagulation/filtration.

Based on these results, coagulation, filtration, and coagulation + filtration tests were conducted to determine the efficiencies of the water treatment processes. Figure 3 shows the turbidity values of the lake water using the coagulation, membrane (PVC + 4 wt % PVP) filtration, and coagulation + filtration processes. From this experiment, the Tanfloc coagulation, membrane filtration, and coagulation + filtrations processes yielded 4.76, 2.33, and 0.95, respectively, turbidity values of the lake water. As shown, the lowest turbidity value was achieved using the coagulation + filtration process on the lake water (Figure 4). In these studies, other parameters such as pH and TDS were also tested on the lake water; however, these parameters were not changed during the experiments. 

Table 4 gives the turbidity, pH, and TDS values of the lake water as a function of the addition of Alum after 1 h and 24 h settlements by gravity. Test results show that after 1 h, the turbidity values of the lake water were reduced to 5.2, 4.8, 3.5, 2.9, and 2.2 NTU at 10, 20, 30, 40, and 50 mg/L Alum dosages, respectively. Turbidity values were further reduced to 3.5, 2.9, 2.2, 2.0, and 1.9 NTU at the same Alum concentrations after one day of settlement time. Likewise, pH and TDS values of these tests remained pretty much the same. Based on the test results, the 50 mg/L of Alum concentration and 1 h duration were selected for the coagulation and filtration tests. 

**Table 4:** Turbidity, pH, and TDS values of lake water as function of Alum addition after 1 h and 24h settlements by gravity.

Experiments	No Coagulation			Coagulation Dosage (mg/L) (1 h)					Coagulation Dosage (mg/L) (1 day)				
	0 h	1 h	24 h	10	20	30	40	50	10	20	30	40	50
Turbidity (NTU)	21.0	17.9	15.8	5.2	4.8	3.5	2.9	2.2	3.5	2.9	2.2	2.0	1.9
pH	8.1			8.1	7.8	7.7	7.5	7.5	8.1	7.8	7.7	7.5	7.5
TDS (ppm)	463			467	465	472	470	472	467	465	472	470	472

**Figure 5:**
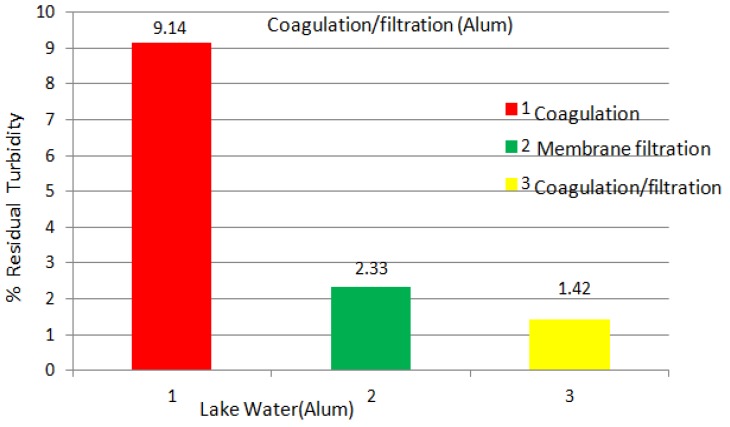
Turbidity values of lake water using Alum coagulation, membrane filtration, and coagulation + filtration processes; coagulation tests conducted using 50 mg/L Alum after 1 h of settlement in flask.

**Figure 6:**
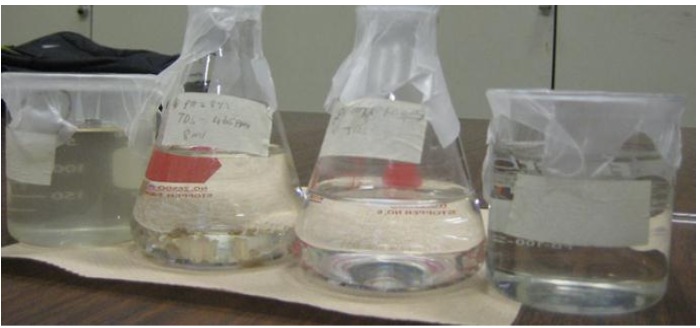
Lake water treatment processes, from left: untreated lake water, Alum coagulation treatment, coagulation/filtration, and direct filtration.

Figure 5 shows the Alum turbidity of the coagulation, membrane filtration, and coagulation + filtration processes on the lake water. From this experiment, the Alum coagulation, membrane filtration, and coagulation + filtration processes yielded 9.14, 2.33, and 1.42, respectively, turbidity values on the lake water, which clearly indicates that the coagulation + filtration process offers the lowest turbidity results on the lake water, as can be seen in Figure 6. These results are comparable to the Tanfloc test results.

#### 2.2.2. Abrasive Jet Water

The same coagulation and filtration tests were conducted on abrasive jet water. The turbidity value of the abrasive jet water was 32 NTU, and after 1 h, this was reduced to 15.0, 13.9, 10.5, 6.7, and 7.5 NTU at 5, 10, 15, 20, and 25 mg/L Tanfloc dosages, respectively (results are not displayed here, but provided elsewhere) [4]. Turbidity values of 20 mg/L at 1 h and 24 h were 6.7 and 6.1, correspondingly, thus, 20 mg/L of Tanfloc and the 1 h settlement time were selected for the coagulation and filtration tests. 

**Figure 7:**
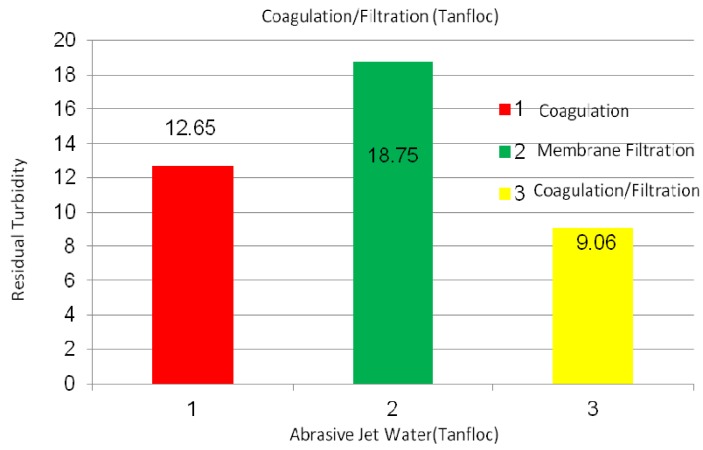
Turbidity values of abrasive jet water using Tanfloc coagulation, membrane filtration, and coagulation + filtration processes; coagulation tests were conducted using 20 mg/L Tanfloc after 1 h of settlement in flask.

Figure 7 shows the Tanfloc coagulation, membrane (PVC + 4 wt % PVP) filtration, and coagulation + filtration processes on the abrasive jet water that includes fiber and resin particles from carbon and glass fiber-reinforced composites. According to the experiment, the Tanfloc coagulation, membrane filtration, and coagulation + filtrations processes yielded 12.65, 18.75, and 9.06 NTU, respectively, turbidity values on the abrasive jet water. The pH values were between 7.7 and 7.4, while the TSD values were between 650 and 660 ppm. As can be seen, the coagulation + filtration process offers the lowest turbidity (cleanest water) results on the abrasive jet water. 

**Figure 8:**
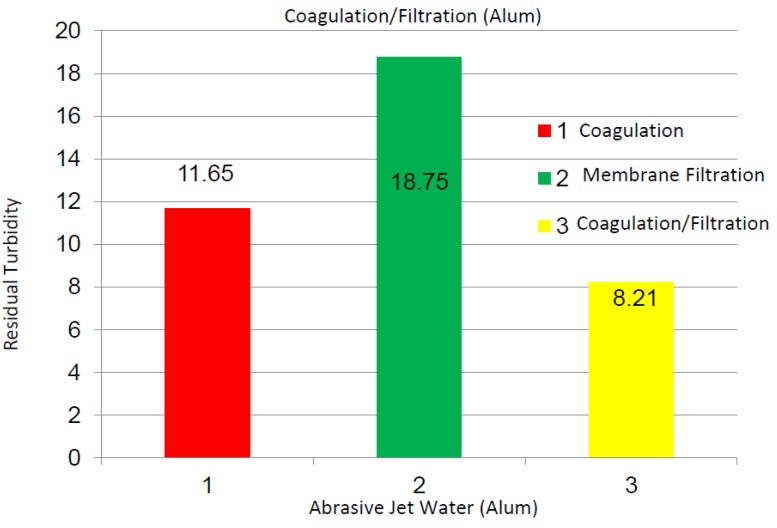
Turbidity values of abrasive jet water using Alum coagulation, membrane filtration, and coagulation + filtration processes; coagulation tests conducted using 50 mg/L Alum after 1 h of settlement in flask.

Alum coagulation tests were applied on the abrasive jet water samples, and 50 mg/L Alum and 1 h settlement time were chosen for the coagulation and filtration tests. Figure 8 shows the turbidity values of the abrasive jet water using the Alum coagulation, membrane filtration, and coagulation + filtration processes. As shown, the Alum coagulation, membrane filtration, and coagulation + filtration processes yielded 11.65, 18.75, and 8.21 NTU, correspondingly, turbidity values on the abrasive jet water. The pH and TDS values were not changed noticeably in these experiments. The residual turbidity values of the membrane filtration for the abrasive water are fairly different when compared to the other water samples. This may be attributed to the contents of this water, such as organic resins and hardeners, inorganic fibers, abrasive particles (garnet and Al oxide), non-uniform particle size distributions, and other contaminations. Overall, the test results indicated that the present filtration system combined with coagulation process effectively worked on the abrasive jet water samples, as well. 

#### 2.2.3. Magnetite Nanoparticles Suspensions

Among the three suspensions, the magnetic nanoparticle suspension has the lowest particle size (10 nm). The purpose of using nanoparticles in water is to find the limit of the present coagulation-filtration system. The turbidity value of the magnetic nanoparticle suspension was 13 NTU and remained the same for 24 h. In the presence of 5, 10, 15, 20, and 25 mg/L of Tanfloc in the suspensions, turbidity values decreased to 6.2, 5.2, 9.1, 9.5, and 9.7 NTU after 1 h, and to 3.1, 3.7, 5.0, 5.5, and 5.5 NTU after 24 h, respectively. Thus, 5 mg/L of Tanfloc and 24 h settlement time were selected for the coagulation and filtration tests. 

**Figure 9:**
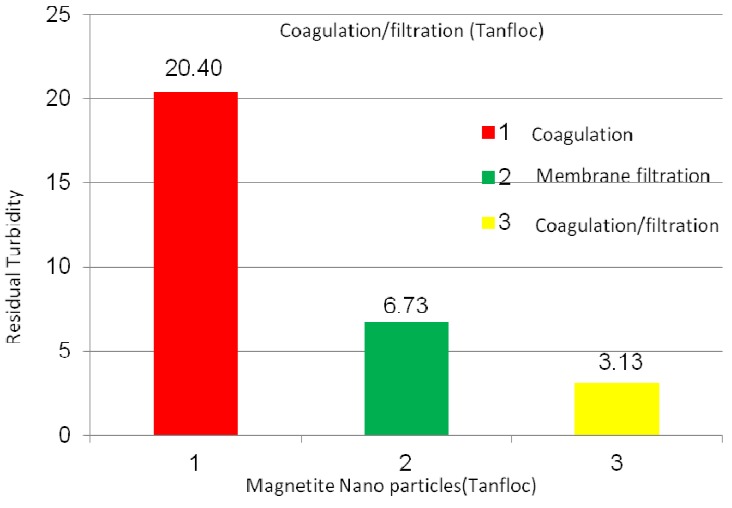
Turbidity values of magnetic nanoparticle suspensions using Tanfloc coagulation, membrane filtration, and coagulation + filtration processes; coagulation tests conducted using 5 mg/L Tanfloc after 24 h of settlement in flask.

Figure 9 shows the Tanfloc coagulation, membrane (PVC + 4 wt % PVP) filtration, and coagulation + filtration processes on the magnetic nanoparticle suspension. The Tanfloc coagulation, membrane filtration, and coagulation + filtration processes yielded 20.4, 6.7, and 3.1 NTU, respectively, turbidity values on the nanoparticle suspensions. The pH values were between 6.6 and 5.9, while the TSD values were between 0.15 and 0.70 ppm. Once again, the coagulation + filtration process displayed the lowest turbidity (cleanest water) values compared to the other options. Some of the agglomerated particles are still at lower size, so the nanofiber membranes mostly captured those agglomerated smaller particles. 

**Figure 10:**
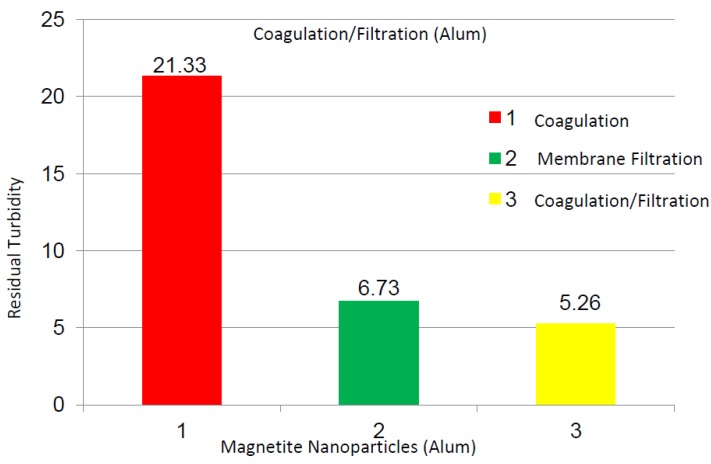
Turbidity values of nanoparticle suspensions using Alum coagulation, membrane filtration, and coagulation + filtration processes; coagulation tests conducted using 10 mg/L Alum after 24 h of settlement in flask.

The Alum coagulation tests were performed on the magnetic nanoparticle suspensions, and 10 mg/L Alum and 24 h of settlement time were chosen for the coagulation and filtration tests. Figure 10 shows the turbidity values of the nanoparticle suspensions using the Alum coagulation, membrane filtration, and coagulation + filtration processes. From Figure 10, the Alum coagulation, membrane filtration, and coagulation + filtration processes yielded 21.33, 6.73, and 5.26 NTU, correspondingly, turbidity values on these samples. Similar pH and TDS values were achieved in these experiments as well. These results indicate that many parameters, such as surface hydrophobicity, coagulation, particle size, and multiple filtration process clearly affect the water quality of the suspensions [19,20,21]. This study is an environmentally friendly approach compared to the other processes and can be extended to enhance the water quality process in many industries.

## 3. Experimental Section

### 3.1. Materials

**Figure 11:**
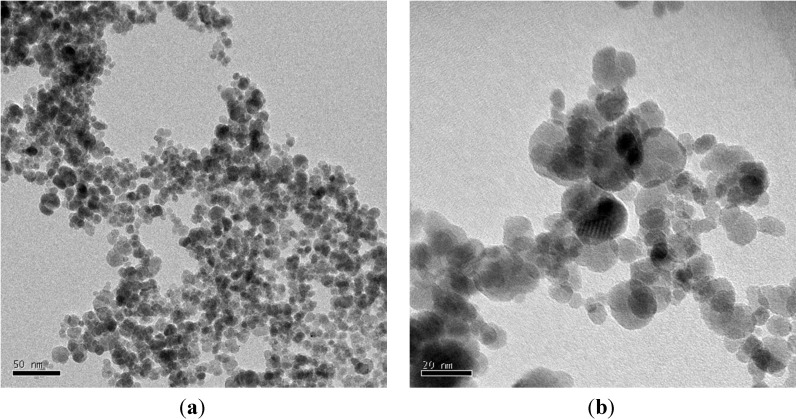
TEM images of magnetite nanoparticles (10 nm) at low (**a**) and high (**b**) magnifications.

PVC, PVP, and dimethylacetamide (DMAc) were purchased from Fisher Scientific. Tanfloc (TANAC, Brazil) and aluminum sulfate (or Alum) (Sigma-Aldrich) were used in the present study to determine the efficiencies of these coagulants on different waste waters. Tanfloc is organic, while Alum is inorganic coagulants. Magnetite nanoparticles were prepared by a co-precipitation process in our laboratory. The details of the process are as follows: 10 mL of 1 M ferric chloride and 2.5 mL of 2 M ferrous chloride were added to a flask containing 50 mL of 1 M hydrochloric acid under stirring conditions at 1200 rpm [4]. After the addition of 25 mL of deionized (D) water to the flask, 50 mL of 3 M ammonium hydroxide was added drop-wise to the solution at room temperature. The resulting solution turned black, indicating the formation of magnetite nanoparticles (~10 nm). The supernatant was decanted using an Nd permanent magnet, and the precipitate was washed several times with DI water. Figure 11 shows the transmission electronic microscopy (TEM) image of magnetite nanoparticles at different magnifications. The purpose for using magnetite nanoparticles was to determine the filtration capabilities (or limits) of the new membrane system.

### 3.2. Methods

The nanomembrane for water filtration was fabricated via the electrospinning process. To fabricate a membrane, first, the PVC was dissolved in DMAc, and then the solution was transferred to a plastic syringe. A high electrostatic field was applied to the polymeric solution to produce electrospun fibers. The total concentration of polymer in the DMAc solution was kept at 15%. To improve the hydrophilicity of the membrane, different weight percentages of PVP (0%, 2%, 3%, 4%, and 5%) were added into the PVC solution prior to electrospinning. However, the total concentration of polymer was still 15 wt %. Throughout the electrospinning process, parameters such as applied voltage (V) and mass flow rate (m) of the polymeric solution were kept constant at 20 kV and 1 mL/h, respectively, while changing the tip to collector distances (D) of 20, 25, and 30 cm. A scanning electron microscope(SEM) was used to determined fiber diameter and morphology of all electrospun membranes. Figure 2 and Figure 3 show SEM images of various-sized electrospun fibers [1,4,18]. 

The contact angle values of the PVC fiber membranes at different concentrations of PVP polymer were measured with an “optical contact angle goniometer” purchased from KSV Instruments Ltd., Model #CAM 100 (Helsinki, Finland). This compact video-based instrument measures contact angles between 1° and 180° with an accuracy of ±1°. Computer software provided by KSV Instruments Ltd., (Helsinki, Finland) precisely records, measures, and takes pictures of the contact angles. For each sample, five contact measurements were read, and the results were averaged. In all coagulation experiments, the changes in parameters such as turbidity, pH, and TDS were considered before and after the coagulation/filtration processes for the lake water, abrasive jet water, and magnetic nanoparticle suspensions. Abrasive jet water consists mainly of fiber and resin particles from carbon and glass fiber-reinforced composites. For the coagulation, membrane filtration, and coagulation + filtration tests, 200 mL of suspended liquids were conditioned in a 250 mL Erlenmeyer flask. Turbidity is the cloudiness of liquids caused by fine suspended particles or molecules [4]. Water quality is usually determined by measuring turbidity with a turbidity meter. The coagulated water samples were tested after both one hour and one day of the coagulation and settlement processes. During the coagulation experiments, the effectiveness in the removal of turbidity is usually achieved after one day of coagulation. For each condition, at least five turbidity readings were conducted, and tests results were averaged. The filtration tests were conducted on different liquids in a 25 inHg vacuum using a Buchner filter at different dosages of Tanfloc (5, 10, 15, 20, and 25 mg/L) and Alum (10, 20, 30, 40, and 50 mg/L) [4].

## 4. Conclusions

Highly purified water can be achieved from various waste water sources by employing electrospun nanofibrous membranes because of their large surface area, flexibility, and small pore size (1–100 nm). Membranes fabricated with 4 wt % PVP in PVC exhibit the desired hydrophilic property (16° water contact angle) that may be ideal for water purification and fouling prevention of many waste waters. These nanofibrous membranes were tested by purifying nine different water samples (lake water, abrasive particles from a water jet cutter, a magnetite solution, and six sub-variants of these three water samples treated by the coagulants Alum and Tanfloc). Water was filtered through the fibrous membranes and collected in a beaker, and three different tests—turbidity, pH, and TDS—were determined. Test results showed that when water suspensions were treated with the coagulants and filtered through the highly hydrophilic membranes, significantly better results were achieved. This can be a low-cost and low-pressure filtration option for the treatment of various waste waters.
